# Signal Source Positioning Based on Angle-Only Measurements in Passive Sensor Networks

**DOI:** 10.3390/s22041554

**Published:** 2022-02-17

**Authors:** Yidi Chen, Linhai Wang, Shenghua Zhou, Renwen Chen

**Affiliations:** 1 State Key Laboratory of Mechanics and Control of Mechanical Structures, Nanjing University of Aeronautics and Astronautics, No. 29, Yudao Street, Nanjing 210016, China; 18001296740@189.cn (Y.C.); rwchen@nuaa.edu.cn (R.C.); 2 National Laboratory of Radar Signal Processing, Xidian University, No. 2 Taibai Road, Xi’an 710071, China; xd2020lhwang@163.com

**Keywords:** passive sensor network, signal localization, data association, angle-only measurements, accuracy analysis

## Abstract

Some passive sensors can measure only directions of arrival of signals, but the real positions of signal sources are often desirable, which can be estimated by combining distributed passive sensors as a network. However, passive observations should be correctly associated first. This paper studies the multi-target data association and signal localization problem in distributed passive sensor networks. With angle-only measurements from distributed passive sensors, multiple lines in a 3-dimensional (3D) scenario can be built and then those that will intersect in a small volume in 3D are classified into the same source. The center of the small volume is taken as an estimate of the signal source position, whose statistical distributions are formulated. If the minimum distance is less than an association threshold, then two lines are considered to be from the same signal source. In numerical results, the impacts of angle measurement accuracy and platform self-positioning accuracy are analyzed, indicating that this method can achieve a prescribed data association rate and a high positioning performance with a low computation cost.

## 1. Introduction

Unlike active sensors such as radars, passive sensors do not transmit signals and thus have no anti-jamming problem [1,2]. However, some passive sensors, such as infrared sensors, photoelectric sensors, electronic counter measurement (ECM) and cameras, can estimate only angles of signal sources. Therefore, their signal source positioning performances are typically poor since they have no accurate range information of signal sources. In order to estimate the positions of signal sources, passive sensors with angle-only observations can be connected with communication links into a network to measure signals sources from different spatial locations. In this case, an algorithm to combine the angle-only observations is needed [3,4,5,6,7]. Compared with the time of arrival (TOA) [8] and the time difference of arrival (TDOA) localization, angle-only localization does not require accurate time synchronization between distributed passive sensors for signal sources with low speeds [6].

In a passive sensor network, there may be multiple signal sources, and before accurate localization, one should first correctly associate observations regarding the same sources [9]. The multi-dimension assignment model is a classical method for data association in passive sensor networks [10,11,12], but it needs the locations of signal sources, which is unavailable before correct data association. A geometry-based localization algorithm for a distributed sensor network is presented in [13], which constructs a test statistic based on the minimal distance between the lines of sight for data association. The measurement errors are considered, but the platform self-positioning errors are not considered.

In this paper, how to perform data association and signal source localization in a 3-dimensional (3D) scenario for distributed passive sensors with only angle measurements is studied. We improve the intersection localization algorithm for passive sensor networks in a multi-target scenario. We first consider the data association problem and then the signal source position estimation problem. The basic concept is to construct a set of lines in a 3D scenario according to angle measurements of signal sources. In data association, measurement lines that will intersect within a small space volume are categorized into the same group. The statistical distribution of the minimal distances of the lines are formulated and the minimum distance between any two observation lines is a random variable, proved to follow the Chi-square distribution. The threshold for correct association is formulated by the misassociation probability; namely, two observations are from the same signal source but are classified into two groups. In the test statistics, not only the measurement errors but also the platform positioning errors are considered, which makes the association performance robust when the platform positioning errors exist.

After data association, observations regarding the same signal sources are grouped, based on which the location of signal sources can be estimated. Three positioning algorithms are considered. It is known that angle measurements are nonlinear functions of coordinates of signal sources. With the Taylor expansion, the least square (LS) algorithm linearizes the nonlinear angle measurements about the target position and then uses the LS method to obtain the target position estimate [6,14,15,16,17,18,19]. In real applications, different passive sensors may obtain observations of different signal-to-noise ratios (SNRs) and then the weighted least squares algorithm (WLS) [6,14] and total least square (TLS) algorithm [20] can be used to obtain a better estimate. Another source location method is the intersection localization algorithm [1,4,5,21,22,23]. The basic concept is that if multiple passive sensors simultaneously measure the signal sources without measurement error, these measurement lines of sight will intersect to the target position. The geometric method and algebraic solution method use this property to estimate the positions of signal sources.

The data association process and target location process of this method are closely combined, which ensures a lower algorithm complexity and a better positioning performance. In numerical results, the improvement of data association and signal-source positioning are analyzed. The impact of the target-sensor geometry on the localization accuracy is also studied, indicating that the localization performance will be better if the lines associated with different observations are perpendicular to each other.

We follow the convention that bold lower and upper case letters denote column vectors and matrices, respectively. A symbol with an upper script *o* denotes the true value. For instance, $a^{o}$ denotes the true value of a. diag(·) with a vector entry denotes a diagonal matrix with the entry vector as diagonal elements. The notation diag($A_{1},A_{2},\ldots ,A_{N}$) stands for the block-diagonal matrix formed by the matrices $A_{1},A_{2},\ldots ,A_{N}$.

## 2. Localization with Angle-Only Passive Sensors

### 2.1. Signal Model of Passive Observations

**Figure 1:**
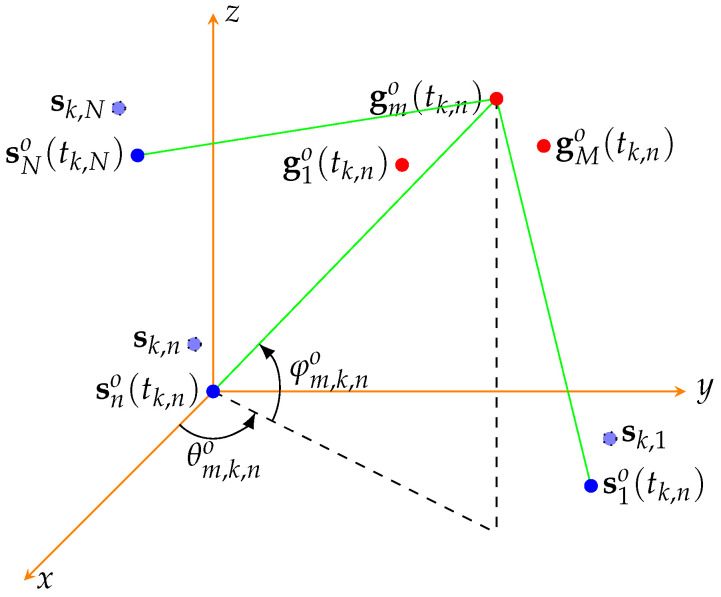
Measurement scenario of the passive sensors.

Consider a passive sensor network with *N* widely separated sensors and *M* targets in the surveillance volume. Assume that a coordinate system is available for all the sensors, such as earth-centered earth-fixed (ECEF) of the World Geodetic System 84 (WGS84). The real position of the *n*th sensor at instant *t* is denoted as $s_{n}^{o}\left(t\right)={\left[x_{n,s}^{o}\left(t\right),y_{n,s}^{o}\left(t\right),z_{n,s}^{o}\left(t\right)\right]}^{T}$, n=1,2,…,N, where ${\left(\cdot \right)}^{T}$ denotes the transpose operation, *t* denotes time, and $x_{n,s}^{o}\left(t\right),y_{n,s}^{o}\left(t\right),z_{n,s}^{o}\left(t\right)$ denote the x,y,z coordinates of the *n*th sensor at instant *t*, respectively. The real position of the *m*th target at instant *t* is denoted by $g_{m}^{o}\left(t\right)={\left[x_{m,g}^{o}\left(t\right),y_{m,g}^{o}\left(t\right),z_{m,g}^{o}\left(t\right)\right]}^{T},m=1,\cdots ,M$, where $x_{m,g}^{o}\left(t\right),y_{m,g}^{o}\left(t\right),z_{m,g}^{o}\left(t\right)$ denote the x,y,z coordinates of the *m*th target at instant *t*, respectively. Assume that there is a self-positioning device to measure the position of each sensor. There are different kinds of instruments that can measure the position of a platform, such as the Global Positioning System (GPS) and inertial sensors. The topology of the passive sensors and targets are shown in Figure 1.

For the *n*th sensor, signals are detected at instants denoted by $t_{k,n},k=1,\cdots ,N_{n}$, where $N_{n}$ denotes the number of observations of the *n*th sensor. At instant $t_{k,n}$, assume that the position of the *n*th sensors is measured as

(1) $$s_{k,n}=s_{n}^{o}\left(t_{k,n}\right)+\Delta s_{n}\left(t_{k,n}\right)={\left[x_{n,s}\left(t_{k,n}\right),y_{n,s}\left(t_{k,n}\right),z_{n,s}\left(t_{k,n}\right)\right]}^{T},k=1,\cdots ,N_{n}$$

where $\Delta s_{n}\left(t_{k,n}\right)$ denotes the sensor self-positioning error. For simplicity, we assume that the positioning errors follow zero mean Gaussian distributions with covariance matrices $C_{k,n}=E\left(\Delta s_{n}\left(t_{k,n}\right)\Delta s_{n}^{T}\left(t_{k,n}\right)\right)$, where E denotes the expectation operation. In practice, the self-positioning error of the sensor is approximately subject to zero-mean Gaussian distribution, so this assumption is reasonable and widely used.

For the angle-only sensors, the observations are directions of the signals and the *l*th signal at instant $t_{k,n}$ is denoted by $\theta_{l,k,n}={\left[\theta_{l,k,n},\varphi_{l,k,n}\right]}^{T}$, where $\left(l,k,n\right)\in M_{k,n}$, $k=1,\cdots ,N_{n}$ and n=1,⋯,N, and $M_{k,n}$ denotes a set of triples of signals detected at the *k*th measurement by the *n*th sensor. Assume that $|M_{k,n}|=M_{k,n}$, where |·| over a set denotes the cardinality of the set. As the existence of miss detection, false alarms and overlapping of signal sources, $M_{k,n}$ may not be equal to *M*. Denote $M_{n}=\cup_{k=1}^{N_{n}}M_{k,n}$ and $A=\cup_{n=1}^{N}M_{n}$, where ∪ denotes the union operation. The total number of observations by *N* sensors is denoted by

(2) $$N_{s}=\left|A\right|=\sum_{n=1}^{N}\sum_{k=1}^{N_{n}}M_{k,n}.$$

At instant $t_{k,n}$, the real position of the *m*th signal source is denoted by

(3) $$g_{m}^{o}\left(t_{k,n}\right)={\left[x_{m,g}\left(t_{k,n}\right),y_{m,g}\left(t_{k,n}\right),z_{m,g}\left(t_{k,n}\right)\right]}^{T},m=1,\cdots ,M.$$

For the *m*th signal source, the real azimuth angle and elevation angle regarding the *n*th sensor can be expressed by

(4) $$\begin{array}{cc} \theta_{m,k,n}^{o} & =\tan^{-1}\left(y_{m,g}^{o}\left(t_{k,n}\right)-y_{n,s}^{o}\left(t_{k,n}\right),x_{m,g}^{o}\left(t_{k,n}\right)-x_{n,s}^{o}\left(t_{k,n}\right)\right)\\ \varphi_{m,k,n}^{o} & =\arctan\left(\left(z_{m,g}^{o}\left(t_{k,n}\right)-z_{n,s}^{o}\left(t_{k,n}\right)\right)/\sqrt{{\left(x_{m,g}^{o}\left(t_{k,n}\right)-x_{n,s}^{o}\left(t_{k,n}\right)\right)}^{2}+{\left(y_{m,g}^{o}\left(t_{k,n}\right)-y_{n,s}^{o}\left(t_{k,n}\right)\right)}^{2}}\right) \end{array}$$

respectively, where $\theta_{m,k,n}^{o}\in \left(-\pi ,\pi \right)$, $\varphi_{m,k,n}^{o}\in \left(-\frac{\pi }{2},\frac{\pi }{2}\right)$, $\tan^{-1}\left(*\right)$ is called the two-argument inverse tangent function [24,25] and arctan(*) is the inverse tangent function. Denote $\theta_{m,k,n}^{o}={\left[\theta_{m,k,n}^{o},\varphi_{m,k,n}^{o}\right]}^{T}$.

Each observation is associated with one of *M* targets or the false alarm indexed by 0, represented by a set M={0,1,⋯,M}. It can be considered as a mapping ψ:A→M. According to our setting, the index set A can be partitioned into M+1 disjoint sets $A_{0},A_{1},\cdots ,A_{M}$ defined by

(5) $$A_{i}=\left\{x|\psi \left(x\right)=i,x\in A\right\},$$

where $A_{0}$ denotes the index of observations corresponding to false alarms, and $A_{m}$ denotes the index set of observations from the *m*th signal source. As a partition of A, we have $A_{i}\cap A_{j}=\emptyset$, i,j∈M, i≠j, and $A=\cup_{i=0}^{M}A_{i}$, where ∩ denotes the intersection operation of sets.

If the *m*th signal source is detected and indexed as the (l,k,n) observation, then the azimuth angle and elevation angle measurements can be written as

(6) $$\begin{array}{cc} \theta_{l,k,n} & ={\left[\theta_{l,k,n},\varphi_{l,k,n}\right]}^{T} \end{array}$$

(7) $$\begin{array}{cc} \; & =\theta_{m,k,n}^{o}+\Delta \theta_{l,k,n} \end{array}$$

(8) $$\begin{array}{cc} \;\theta_{l,k,n} & =\theta_{m,k,n}^{o}+\Delta \theta_{l,k,n} \end{array}$$

(9) $$\begin{array}{cc} \;\varphi_{l,k,n} & =\varphi_{m,k,n}^{o}+\Delta \varphi_{l,k,n} \end{array}$$

(10) $$\begin{array}{cc} \;\Delta \theta_{l,k,n} & ={\left[\Delta \theta_{l,k,n},\Delta \theta_{l,k,n}\right]}^{T} \end{array}$$

where $\Delta \theta_{l,k,n}$ and $\Delta \varphi_{l,k,n}$ represent the measurement noise of the azimuth angle and elevation angle, respectively. For simplicity, we assume that observation noises $\Delta \theta_{l,k,n}$ and $\Delta \varphi_{l,k,n},l=1,\cdots ,M_{k,n},k=1,\cdots ,N_{n},n=1,\cdots ,N$ are statistically independent and follow zero-mean Gaussian distribution by assumption. The covariance matrices of Δθ are denoted by $C_{l,k,n}=E\left(\Delta \theta \Delta \theta^{T}\right)\in C^{2\times 2}$, namely $\Delta \theta ∼N(0,C_{l,k,n})$, which is typically affected by the SNR of the signal, where $N(0,C_{l,k,n})$ denotes the zero-mean Gaussian distribution with mean 0 and covariance matrix $C_{l,k,n}$. It should be noted that modeling the angle measurement noise as a zero-mean Gaussian distribution is a commonplace assumption.

### 2.2. The Distance of Observation Lines

The angle-only observations provide information on the directions of signal sources, and in theory, the directions can be expressed by 3D lines. Therefore, it is important to study the properties of the lines. Consider a simple scenario where the target can be deemed to be static when the observations are recorded and the location of the *m*th signal is simplify denoted by $g_{m}^{o}$. For passive observations, each observation, say (l,k,n), will contribute a line in space and the line can be written as $L_{l,k,n}$

(11) $$\begin{array}{c} L_{l,k,n}:x=s_{k,n}+\alpha_{l,k,n}e_{l,k,n},\alpha_{l,k,n}\in R \end{array}$$

where $\alpha_{l,k,n}$, a parameter indicating the distance to the origin $s_{k,n}$, $e_{n,m}={\left[e_{l,k,n,x},e_{l,k,n,y},e_{l,k,n,z}\right]}^{T}\in R^{3\times 1}$, is the normalized direction vector associated with the angle observation $\theta_{l,k,n}$, and

(12) $$\begin{array}{cc} e_{l,k,n,x} & =\cos\left(\theta_{l,k,n}\right)\cos\left(\varphi_{l,k,n}\right) \end{array}$$

(13) $$\begin{array}{cc} e_{l,k,n,y} & =\sin\left(\theta_{l,k,n}\right)\cos\left(\varphi_{l,k,n}\right) \end{array}$$

(14) $$\begin{array}{cc} e_{l,k,n,y} & =\sin(\varphi_{l,k,n}).\; \end{array}$$

It can be seen that the subscripts of the denotations are complicated. Therefore, for two observations $i,j\in A_{m}$, we simplify the expression of lines by

(15) $$\begin{array}{cc} L_{i,m}:x & =s_{i}+\alpha_{i,m}e_{i,m} \end{array}$$

(16) $$\begin{array}{cc} L_{j,m}:x^{\prime } & =s_{j}+\alpha_{j,m}e_{j,m} \end{array}$$

where the locations of two sensors regarding the two observations are denoted by $s_{i},s_{j}$, $\alpha_{i,m},\alpha_{j,m}$ are two scalars indicating the distances to two origins, and $e_{i,m}$ and $e_{j,m}$ are two normalized vectors associated with two observations.

The difference between two points over the two lines are

(17) $$\begin{array}{cc} \Rightarrow \;x^{\prime }-x & =s_{j}-s_{i}+\alpha_{j,m}e_{j,m}-\alpha_{i,m}e_{i,m} \end{array}$$

(18) $$\begin{array}{cc} \; & =s_{j}-s_{i}+\left(-e_{i,m},e_{j,m}\right)\alpha , \end{array}$$

where

(19) $$\alpha =\left[\begin{array}{c} \alpha_{i,m}\\ \alpha_{j,m} \end{array}\right].$$

Without measurement error, then there will be two $\alpha_{i,m}$ and $\alpha_{j,m}$ such that $x^{\prime }-x=0$, i.e.,

(20) $$g_{m}=s_{i}+\alpha_{i,m}e_{i,m}=s_{j}+\alpha_{j,m}e_{j,m}.$$

In general, due to inevitable measurement errors, the lines even regarding the same signal source may not coincide to each other. Therefore, we calculate minimal distance between those lines. The distance between two points over two lines can be expressed by

(21) $$\begin{array}{cc} d= & ∥x^{\prime }{-x∥}^{2}\\ = & ∥s_{j}-s_{i}+\left(-e_{i,m},e_{j,m}\right){\alpha ∥}^{2}\\ = & \alpha^{T}E_{i,j}^{T}E_{i,j}\alpha +2\alpha^{T}E_{i,j}^{T}\left(s_{j}-s_{i}\right)+{\left(s_{j}-s_{i}\right)}^{T}\left(s_{j}-s_{i}\right) \end{array}$$

where ∥·∥ denotes the $\ell_{2}$-norm, $\alpha ={\left[\alpha_{i,m},\alpha_{j,m}\right]}^{T}$, and $E_{i,j}=\left[-e_{i,m},e_{j,m}\right]$. For simplicity, the distance *d* is actually the squared distance, instead of the distance.

In particular, if $e_{i,m}=e_{j,m}$, namely two lines are parallel to each other, then

(22) $$d=∥s_{j}-s_{i}+\left(\alpha_{j,m}-\alpha_{i,m}\right)e_{i,m}{∥}^{2},$$

and the minimal distance between points in two lines will be achieved if

(23) $$\alpha_{j,m}-\alpha_{i,m}=e_{i,m}^{T}\left(s_{i}-s_{j}\right).$$

It can be proved that the minimal distance is

(24) $$d=∥\left(e_{i,m}e_{i,m}^{T}-I\right)\left(s_{i}-s_{j}\right){∥}^{2}.$$

If $e_{i,m}\neq e_{j,m}$ or $|E_{i,j}|\neq 0$, the distance is a second order function of $\alpha_{i,m}$ and $\alpha_{j,m}$, thus the minimal value of *d* is unique, where |·| over a matrix denotes the determinant of the input matrix. To obtain the minimal value, we take a derivative of *d* with respect to α,

(25) $$\begin{array}{cc} \frac{dd}{d\alpha } & ={\left[\frac{dd}{d\alpha_{i,m}},\frac{dd}{d\alpha_{j,m}}\right]}^{T} \end{array}$$

(26) $$\begin{array}{cc} \; & =2E_{i,j}^{T}E_{i,j}\alpha +2E_{i,j}^{T}\left(s_{j}-s_{i}\right) \end{array}$$

where

(27) $$\begin{array}{cc} \frac{dd}{d\alpha_{i,m}} & =-2e_{i,m}^{T}\left(s_{j}-s_{i}+E\alpha \right) \end{array}$$

(28) $$\begin{array}{cc} \frac{dd}{d\alpha_{j,m}} & =2e_{j,m}^{T}\left(s_{j}-s_{i}+E\alpha \right). \end{array}$$

Let the derivative be zero and then we obtain a solution

(29) $$\frac{dd}{d\alpha }=0\Rightarrow \;E_{i,j}^{T}E_{i,j}\alpha =-E_{i,j}^{T}\left(s_{j}-s_{i}\right).$$

Under the assumption that $|E_{i,j}|\neq 0$, $R_{i,j}=E_{i,j}^{T}E_{i,j}$ has a reverse matrix and then the solution can be immediately obtained by

(30) $$\alpha_{\mathrm{opt}}=-{\left(E_{i,j}^{T}E_{i,j}\right)}^{-1}E_{i,j}^{T}\left(s_{j}-s_{i}\right).$$

The minimal distance can be expressed by

(31) $$\begin{array}{cc} d_{\min} & ={\left(s_{j}-s_{i}\right)}^{T}\left(I-E_{i,j}R_{i,j}^{-1}E_{i,j}^{T}\right)\left(s_{j}-s_{i}\right) \end{array}$$

(32) $$\begin{array}{cc} \; & =∥\left(I-E_{i,j}R_{i,j}^{-1}E_{i,j}^{T}\right)\left(s_{j}-s_{i}\right){∥}^{2}.\; \end{array}$$

In particular, if

(33) $$E_{i,j}R_{i,j}^{-1}E_{i,j}^{T}\left(s_{j}-s_{i}\right)=s_{j}-s_{i},$$

namely, $s_{j}-s_{i}$ is an eigenvector of $E_{i,j}R_{i,j}^{-1}E_{i,j}^{T}$ and the eigenvalue is 1, then

(34) $$d_{\min}=0.$$

It means that two lines will intersect at a point. Without measurement errors, observations regarding the same target will form lines intersecting at the target position.

In practice, the real mapping ψ should be estimated through an association algorithm. Due to measurement errors, the estimated mapping may not be correct, and then, the positioning error may raise. Therefore, an accurate data association method is important, which will be studied subsequently.

## 3. Data Association Based on Minimal Distance

### 3.1. Data Association Model

In order to perform data association, we first need to build the statistical model of the minimal distance of the observation lines. Because of both the platform positioning error $\Delta s_{i},\Delta s_{j}$ and the angle measurement error $\Delta \theta_{i,m}$ and $\Delta \theta_{j,m},i,j\in A_{m}$, the minimal distance $d_{\min}$ is not zero and follows a certain distribution depending on the measurement errors, where $\Delta s_{i}$, $\Delta s_{j}$ denote the sensor self-positioning errors of the *i*th and the *j*th observations, respectively, and $\Delta \theta_{i,m},\Delta \theta_{j,m}$ denote the angle measurement error of the *i*th and the *j*th observations on the *m*th target.

In practice, the *i*th and the *j*th observations may belong to the same target or not, and the data association problem is treated as a test to make a decision here. For i,j∈A, the data association problem for a target can be formulated as the following hypothesis problem

(35) $$\left\{\begin{array}{cc} H_{0}:d_{\min}=0 & \psi (i)=\psi (j)\\ H_{1}:d_{\min}>0 & \psi (i)\neq \psi (j). \end{array}\right.$$

To determine the statistical distribution of $d_{\min}$ under the $H_{0}$ hypothesis, we first define

(36) $$K_{i,j}=I-E_{i,j}R_{i,j}^{-1}E_{i,j}^{T}$$

and then

(37) $$d_{\min}={∥K_{i,j}\left(s_{j}-s_{i}\right)∥}^{2}.$$

Next, we discuss the eigenvalues of $K_{i,j}$. It can be proved that $K_{i,j}^{T}K_{i,j}=K_{i,j}$ and $K_{i,j}^{T}=K_{i,j}$. Therefore, $K_{i,j}$ is a positive semi-definite matrix and its possible eigenvalues are either 0 or 1. As $K_{i,j}$ is not an all-zero matrix, then there is at least an eigenvalue of 1.

The trace of the matrix K(i,j) satisfies

(38) $$\begin{array}{cc} \mathrm{tr}(K_{i,j}) & =\mathrm{tr}(I-E_{i,j}R_{i,j}^{-1}E_{i,j}^{T}) \end{array}$$

(39) $$\begin{array}{cc} \; & =\mathrm{tr}\left(I\right)-\mathrm{tr}\left(E_{i,j}R_{i,j}^{-1}E_{i,j}^{T}\right) \end{array}$$

(40) $$\begin{array}{cc} \; & =3-\mathrm{tr}(E_{i,j}^{T}E_{i,j}R_{i,j}^{-1}) \end{array}$$

(41) $$\begin{array}{cc} \; & =3-\mathrm{tr}(R_{i,j}R_{i,j}^{-1}) \end{array}$$

(42) $$\begin{array}{cc} \; & =3-\mathrm{tr}(I_{2}\in R^{2\times 2})=1 \end{array}$$

where tr(·) with a matrix input denotes the trace of the matrix.

Therefore, it can be inferred that three eigvenvalues of $K_{i,j}$ are 1,0,0 and the rank of $K_{i,j}$ is 1. In other words, $K_{i,j}$ can be written as

(43) $$K_{i,j}=e_{s}e_{s}^{T}$$

where $e_{s}$ is a unity direction vector perpendicular to both $e_{i}$ and $e_{j}$, defined by

(44) $$e_{s}=e_{i}\times e_{j}/\sqrt{1-{\left(e_{i}^{T}e_{j}\right)}^{2}}$$

and $e_{i}$ and $e_{j}$ denote the unity direction vectors associated with the *i*th and the *j*th observations, respectively.

With this fact, the minimal distance can be rewritten as

(45) $$d_{\min}={\left|e_{s}^{T}\left(s_{j}-s_{i}\right)\right|}^{2}.$$

In practice, both $s_{j}-s_{i}$ and $e_{s}$ may be inaccurate. A statistical distribution is necessary to determine the impact of the measurement errors. In order to formulate the statistical distribution of $d_{\min}$ under the $H_{0}$ hypothesis, we define

(46) $$r=e_{s}^{T}\left(s_{j}-s_{i}\right)$$

and its relationship to $d_{\min}$ is $d_{\min}={\left|r\right|}^{2}$.

The minimal distance varies with a total of 10 parameters; namely $\theta_{i,m},\theta_{j,m},s_{i,m}$ and $s_{j,m}$, and the corresponding partial derivatives can be written as

(47) $$\begin{array}{cc} \frac{\partial r}{\partial \theta_{i,m}} & ={\left[\frac{\partial r}{\partial \theta_{i,m}},\frac{\partial r}{\partial \varphi_{i,m}}\right]}^{T} \end{array}$$

(48) $$\begin{array}{cc} \frac{\partial r}{\partial \theta_{j,m}} & ={\left[\frac{\partial r}{\partial \theta_{j,m}},\frac{\partial r}{\partial \varphi_{j,m}}\right]}^{T} \end{array}$$

(49) $$\begin{array}{cc} \frac{\partial r}{\partial s_{i}} & =-e_{s}\; \end{array}$$

(50) $$\begin{array}{cc} \frac{\partial r}{\partial s_{j}} & =e_{s}.\; \end{array}$$

Consequently, under the $H_{0}$ hypothesis, with denotation $v={\left[\theta_{i,m}^{T},\theta_{j,m}^{T},s_{i,m}^{T},s_{j,m}^{T}\right]}^{T}$, we have an approximation

(51) $$\begin{array}{cc} r & \approx \frac{\partial r}{\partial \theta_{i,m}^{T}}\Delta \theta_{i,m}+\frac{\partial r}{\partial \theta_{j,m}^{T}}\Delta \theta_{j,m}+\frac{\partial r}{\partial s_{i}^{T}}\Delta s_{i,m}+\frac{\partial r}{\partial s_{j}^{T}}\Delta s_{j,m} \end{array}$$

(52) $$\begin{array}{cc} \; & =\frac{\partial r}{\partial v^{T}}\Delta v\; \end{array}$$

where $\Delta v={\left[\Delta \theta_{i,m}^{T},\Delta \theta_{j,m}^{T},\Delta s_{i,m}^{T},\Delta s_{j,m}^{T}\right]}^{T}$, and

(53) $$\frac{\partial r}{\partial v^{T}}=\left[\frac{\partial r}{\partial \theta_{i,m}^{T}},\frac{\partial r}{\partial \theta_{j,m}^{T}},\frac{\partial r}{\partial s_{i}^{T}},\frac{\partial r}{\partial s_{j}^{T}}\right].$$

Under the assumption that the measurement errors $\Delta \theta_{i,m},\Delta \theta_{j,m},\Delta s_{i,m},\Delta s_{j,m}$ are statistically independent of each other and follow a zero-mean Gaussian distribution, *r* follows the Gaussian distribution and then $d_{\min}$ follows the central weighted Chi-square distribution with 1 degree of freedom, namely $d_{\min}∼\chi_{1}^{2}\left(\lambda \right)$, where λ is the variance

(54) $$\lambda =E\left(\frac{\partial r}{\partial v^{T}}\Delta v\Delta v^{T}\frac{\partial r}{\partial v}\right)=\frac{\partial r}{\partial v^{T}}C_{v}\frac{\partial r}{\partial v}$$

and

(55) $$C_{v}=E\left(\Delta v\Delta v^{T}\right).$$

The probability density function (PDF) and cumulative distribution function (CDF) can be written as

(56) $$\begin{array}{cc} p_{d_{\min}}\left(d\right) & =\frac{1}{2\lambda \sqrt{\pi }}{\left(\frac{d}{\lambda }\right)}^{-\frac{1}{2}}\exp\left(-\frac{d}{2\lambda }\right),d\geq 0 \end{array}$$

(57) $$\begin{array}{cc} F_{d_{\min}}\left(d\right) & =\frac{1}{\sqrt{\pi }}\gamma \left(\frac{d}{2\lambda },\frac{1}{2}\right),d\geq 0\; \end{array}$$

respectively, where Γ(·) denotes the Gamma function and γ(·,·) denotes the incomplete Gamma function. If the decision rule is to keep the misassociation probability $P(H_{1}|H_{0})$ as a constant, say $p_{f}$, then the decision threshold can be obtained as

(58) $$\begin{array}{c} \rho =2\lambda \gamma^{-1}\left(\sqrt{\pi }\left(1-p_{f}\right),\frac{1}{2}\right) \end{array}$$

where $\gamma^{-1}\left(\cdot ,\frac{1}{2}\right)$ denotes the inverse incomplete Gamma function with $\frac{1}{2}$ a degree of freedom.

Therefore, the key is to derive the variance λ. In practice, as the measurements are carried out by different sensors, it is reasonable to assume that the measurement errors are statistically independent of each other. In this case, $C_{v}$ is a block diagonal matrix and the variance can be formulated conveniently.

### 3.2. Measurement Errors and Association Threshold

For simplicity, we first consider the self-positioning error $\Delta s_{i},\Delta s_{j}$, whose covariance matrices are assumed to be

(59) $$\begin{array}{cc} C_{i,s} & =E(\Delta s_{i}\Delta s_{i}^{T}) \end{array}$$

(60) $$\begin{array}{cc} \;C_{j,s} & =E(\Delta s_{j}\Delta s_{j}^{T}). \end{array}$$

Consequently, from (49) and (50), the variances due to the two terms are

(61) $$\begin{array}{cc} \lambda_{i,s} & =E\left(\frac{\partial r}{\partial s_{i}}\Delta s_{i,m}\Delta s_{i,m}^{T}{\left(\frac{\partial r}{\partial s_{i}}\right)}^{T}\right)=e_{s}^{T}C_{i,s}e_{s} \end{array}$$

(62) $$\begin{array}{cc} \;\lambda_{j,s} & =E\left(\frac{\partial r}{\partial s_{j}}\Delta s_{j,m}\Delta s_{j,m}^{T}{\left(\frac{\partial r}{\partial s_{j}}\right)}^{T}\right)=e_{s}^{T}C_{j,s}e_{s}. \end{array}$$

Next, we consider the variance caused by the angle measurement error $\Delta \theta_{i,m}$ and $\Delta \theta_{j,m}$. Assume that the covariance matrices of $\Delta \theta_{i,m}$ and $\Delta \theta_{j,m}$ are

(63) $$\begin{array}{cc} C_{i,\theta } & =E(\Delta \theta_{i,m}\Delta \theta_{i,m}^{T}) \end{array}$$

(64) $$\begin{array}{cc} C_{j,\theta } & =E(\Delta \theta_{j,m}\Delta \theta_{j,m}^{T}) \end{array}$$

respectively. The variances caused by the two terms are

(65) $$\begin{array}{cc} \lambda_{i,\theta }= & E\left(\frac{\partial r}{\partial \theta_{i,m}^{T}}\Delta \theta_{i,m}\Delta \theta_{i,m}^{T}\frac{\partial r}{\partial \theta_{i,m}}\right)=\frac{\partial r}{\partial \theta_{i,m}^{T}}E\left(\Delta \theta_{i,m}\Delta \theta_{i,m}^{T}\right)\frac{\partial r}{\partial \theta_{i,m}}=\frac{\partial r}{\partial \theta_{i,m}^{T}}C_{i,\theta }\frac{\partial r}{\partial \theta_{i,m}} \end{array}$$

(66) $$\begin{array}{cc} \lambda_{j,\theta }= & E\left(\frac{\partial r}{\partial \theta_{j,m}^{T}}\Delta \theta_{j,m}\Delta \theta_{j,m}^{T}\frac{\partial r}{\partial \theta_{j,m}}\right)=\frac{\partial r}{\partial \theta_{j,m}^{T}}E\left(\Delta \theta_{j,m}\Delta \theta_{j,m}^{T}\right)\frac{\partial r}{\partial \theta_{j,m}}=\frac{\partial r}{\partial \theta_{j,m}^{T}}C_{j,\theta }\frac{\partial r}{\partial \theta_{j,m}} \end{array}$$

It can be seen that the distance is not a linear function of θ. As a cross product of $e_{i,m}$ and $e_{j,m}$, $e_{s}$ is a complicated function. From (47) and (48), $\frac{\partial r}{\partial \theta_{i,m}}$ and $\frac{\partial r}{\partial \theta_{j,m}}$ are proved in Appendix A.

Under these assumptions, $C_{v}=\mathrm{diag}\left(C_{i,\theta },C_{j,\theta },C_{i,s},C_{i,s}\right)$. Consequently, the variance λ can be expressed by

(67) $$\lambda =\lambda_{i,s}+\lambda_{j,s}+\lambda_{i,\theta }+\lambda_{j,\theta }.$$

It should be noted that under the $H_{1}$ hypothesis, the distance may be arbitrary, and for simplicity, we assume that the distance is uniformly distributed over the surveillance volume. In this case, we can determine whether two observations are from the same target by the following decision rule

(68) $$\begin{array}{c} d_{\min}\overset{H_{1}}{\underset{H_{0}}{≷}}\rho . \end{array}$$

The association algorithm is a mapping $\psi^{\prime }:A\to M$, which partitions A into M+1 groups and this mapping may disagree with real ψ due to inevitable errors. In practice, some knowledge about the targets of interest may be available and thus can be used for better performance. For instance, if only targets on the ground are of interest, then we may use this information and discard observations in which the cross points are obviously apart from the ground, which will be studied in the future.

### 3.3. Localization Algorithms

With mapping $\psi^{\prime }$, we obtain another partition $A=\cup_{m=0}^{M}A_{m}^{\prime }$, where $A_{m}^{\prime }=\left\{x|\psi^{\prime }\left(x\right)=m,x\in A\right\}$. For observations in $A_{m}^{\prime }$, we can conduct signal source positioning, and three target positioning estimation methods will be introduced subsequently.

We first consider the intersection method. In presence of measurement errors, we often have $d_{\min}\neq 0$ even if two observations are from the same target. In this case, over two lines, the two points with minimal distance are

(69) $$\begin{array}{cc} x_{i} & =s_{i}+\alpha_{\mathrm{opt}}\left(1\right)e_{i,m} \end{array}$$

(70) $$\begin{array}{cc} x_{j} & =s_{j}+\alpha_{\mathrm{opt}}\left(2\right)e_{j,m}. \end{array}$$

In this case, it is reasonable to take the middle of two points as the estimate of the signal position, namely

(71) $$\begin{array}{cc} {\hat{g}}_{i,j} & =\frac{1}{2}\left(x_{i}+x_{j}\right)\\ \; & =\frac{1}{2}\left(s_{i}+s_{j}\right)-\frac{1}{2}\left(e_{i,m},e_{j,m}\right)R_{i,j}^{-1}E_{i,j}^{T}\left(s_{j}-s_{i}\right). \end{array}$$

With more observations available, there will be an estimate of the target location for each observation pair, and a simple estimate of the target location can be expressed by their average, namely

(72) $${\hat{g}}_{m}=\frac{1}{N_{m}\left(N_{m}-1\right)}\sum_{i,j\in A_{m}}{\hat{g}}_{i,j}$$

where $N_{m}$ denotes the number of observations associated with the *m*th signal source.

In practice, another widely used localization algorithm is the LS algorithm. It stems from the delta method concerned in [26]. From (4), the *i*th angle observation denoted by $\theta_{i,m}$ actually contributes a geometric relationship, which can be expressed by

(73) $$G_{i}g_{m}=G_{i}s_{i},i\in A_{m}$$

where

(74) $$\begin{array}{cc} G_{i} & =\left[\begin{array}{ccc} \sin\theta_{i,m} & -\cos\theta_{i,m} & 0\\ \cos\theta_{i,m}\sin\varphi_{i,m} & \sin\theta_{i,m}\sin\varphi_{i,m} & -\cos\varphi_{i,m} \end{array}\right]. \end{array}$$

In (74), we used the equality

(75) $$\begin{array}{cc} \; & \left(x_{m,g}^{o}-x_{i,s}^{o}\right)\cos\theta_{i,m}^{o}+\left(y_{m,g}^{o}-y_{i,s}^{o}\right)\sin\theta_{i,m}^{o} \end{array}$$

(76) $$\begin{array}{cc} = & \sqrt{{\left(x_{m,g}^{o}-x_{i,s}^{o}\right)}^{2}+{\left(y_{m,g}^{o}-y_{i,s}^{o}\right)}^{2}}\\ = & d_{i,m}^{o}\cos\varphi_{i,m}^{o} \end{array}$$

where the value $d_{i,m}^{o}$ is the true range of $g_{m}^{o}$ to $s_{i}^{o}$ and can be expressed by $d_{i,m}^{o}=∥g_{m}^{o}-s_{i}^{o}∥$.

Next, we can combine the observations into an equation

(77) $$Gg_{m}=y$$

where

(78) $$\begin{array}{cc} G=\left[\begin{array}{c} G_{1}\\ \vdots \\ G_{M_{m}} \end{array}\right],\;y & =\left[\begin{array}{c} G_{1}s_{1}\\ \vdots \\ G_{M_{m}}s_{M_{m}} \end{array}\right]. \end{array}$$

It should be noted that after the data association operation, the number of samples associated with a target may not agree with the real number. For simplicity, we still use $M_{m}=\left|A_{m}^{\prime }\right|$ to denote the number of observations associated with the *m*th target.

With $M_{m}$ observations, there are in total $2M_{m}$ equations and three unknown parameters in $g_{m}$. Therefore, as if $M_{m}\geq 2$, we can use the LS method to obtain an optimal solution in the sense of the mean square error, as

(79) $$\begin{array}{c} {\hat{g}}_{m}={\left(G^{T}G\right)}^{-1}Gy. \end{array}$$

In practice, different observations may have different SNRs, and then, the WLS algorithm can also be used in this framework. Then, the angle measurement error and sensor positioning error are equally weighted in the process of the LS algorithm. Assume that the distribution of the angle measurement error and the positioning error of sensors are known a priori. With this information, we can impose different weights over the observations, which is the WLS algorithm.

With the following approximations

(80) $$\begin{array}{cc} \sin\theta_{i,m} & \approx \sin\left(\theta_{i,m}^{o}\right)+\Delta \theta_{i,m}^{T}\cos\theta_{i,m}^{o} \end{array}$$

(81) $$\begin{array}{cc} \cos\theta_{i,m} & \approx \cos\left(\theta_{i,m}^{o}\right)-\Delta \theta_{i,m}^{T}\sin\theta_{i,m}^{o} \end{array}$$

we can put (80) and (81) into (73) and then write (73) as

(82) $$\begin{array}{cc} \epsilon_{i} & =G_{i}\left(g_{m}^{o}-s_{i}\right) \end{array}$$

where

(83) $$\begin{array}{cc} \epsilon_{i} & ={\left[\epsilon_{i,1},\epsilon_{i,2}\right]}^{T} \end{array}$$

(84) $$\begin{array}{cc} \;\epsilon_{i,1} & =\Delta \theta_{i,m}d_{i,m}^{o}\cos\varphi_{i,m}^{o}+a_{i,m}^{oT}\Delta s_{i} \end{array}$$

(85) $$\begin{array}{cc} \;\epsilon_{i,2} & =\Delta \varphi_{i,m}d_{i,m}^{o}+b_{i,m}^{oT}\Delta s_{i} \end{array}$$

(86) $$\begin{array}{cc} \;a_{i,m}^{o} & ={\left[-\sin\theta_{i,m}^{o},\cos\theta_{i,m}^{o},0\right]}^{T} \end{array}$$

(87) $$\begin{array}{cc} b_{i,m}^{o} & ={\left[-\cos\theta_{i,m}^{o}\sin\varphi_{i,m}^{o},-\sin\theta_{i,m}^{o}\sin\varphi_{i,m}^{o},\cos\varphi_{i,m}^{o}\right]}^{T}. \end{array}$$

In (84), we have used the equality (76). In (85), we have used the equality

(88) $$\begin{array}{cc} \left(x_{m,g}^{o}-x_{i,s}^{o}\right)\sin\theta_{i,m}^{o}-\left(y_{m,g}^{o}-y_{i,s}^{o}\right)\cos\theta_{i,m}^{o} & =0 \end{array}$$

(89) $$\begin{array}{cc} {\left(g_{m}^{o}-s_{i}^{o}\right)}^{T}{\left[\cos\theta_{i,m}^{o}\cos\varphi_{i,m}^{o},\sin\theta_{i,m}^{o}\cos\varphi_{i,m}^{o},\sin\varphi_{i,m}^{o}\right]}^{T} & =d_{i,m}^{o}\; \end{array}$$

which can be easily verified in the angle measurement equations.

The vector $\epsilon_{i}$ can be rewritten as

(90) $$\epsilon_{i}=B_{i}^{o}\delta_{i}$$

where

(91) $$\begin{array}{cc} B_{i}^{o} & =\left[\begin{array}{ccc} d_{i,m}^{o}\cos\varphi_{i,m}^{o} & 0 & a_{i,m}^{o,T}\\ 0 & d_{i,m}^{o} & b_{i,m}^{o,T} \end{array}\right] \end{array}$$

(92) $$\begin{array}{cc} \delta_{i} & ={\left[\Delta \theta_{i,m},\Delta \varphi_{i,m},\Delta s_{i}^{T}\right]}^{T}.\; \end{array}$$

Under the assumption that the self-positioning error and the angle measurement error are decorrelated, it can be proven that the covariance matrix of $\epsilon_{i}$ can be written as

(93) $$Q_{n}=E\left(\epsilon \epsilon^{T}\right)=\mathrm{diag}\left(C_{n,\theta },C_{n,s}\right).$$

Putting (90) into (82) and combining the observations into an equation yield

(94) $$\begin{array}{c} B^{o}\delta =Gg_{m}^{o}-y. \end{array}$$

where

(95) $$\begin{array}{cc} B^{o}=\left[\begin{array}{c} B_{1}^{o}\\ \vdots \\ B_{M_{m}}^{o} \end{array}\right],\;\delta & =\left[\begin{array}{c} \delta_{1}\\ \vdots \\ \delta_{M_{m}} \end{array}\right]. \end{array}$$

In the WLS algorithm, the goal is to minimize the objective function $J(g_{m})$ as

(96) $$\begin{array}{c} J\left(g_{m}\right)={\left(Gg_{m}-y\right)}^{T}W\left(Gg_{m}-y\right) \end{array}$$

where W is the weighting matrix with the expression

(97) $$\begin{array}{c} W=E{\left[B^{o}\delta \delta^{T}B^{oT}\right]}^{-1}={\left(B^{o}QB^{oT}\right)}^{-1} \end{array}$$

and Q is the error covariance matrix with an expression $Q=\mathrm{diag}(Q_{1},Q_{2},\ldots ,Q_{N})$.

The variable $g_{m}$ to minimize the objective function $J(g_{m})$ can be calculated by the least square method and the estimate of the target positioning can be expressed by

(98) $$\begin{array}{c} {\hat{g}}_{m}={\left(G^{T}WG\right)}^{-1}G^{T}Wy \end{array}$$

which is the WLS algorithm.

In WLS, the covariance matrices of different observations should be known a priori. If the observations can be deemed to have close SNRs, then one can simply use the LS algorithm. If the covariance matrices are not known with certain accuracy, some performance loss may occur, which should be analyzed with numerical analysis.

## 4. Numerical Results

We first study the localization performance in the presence of a platform of self-positioning errors and angle measurement errors. Then, the data association performance will be analyzed, followed by the analysis of the impact of sensor-target geometry.

Consider a scenario where two sensors are installed on two aircraft and three targets of interest are in the scope. Assume that two aircraft move at the same speed. The parameter settings are shown in Table 1. For simplicity, we assume that two passive sensors collect their observations on the same instants, namely, $t_{k,i}=t_{k,j}$ for all *k* and i,j∈{1,⋯,N}. Meanwhile, the sampling interval is 0.1 s and the simulation runs for 10 s. Assume that the covariance matrices of angle measurement error for all sensors and all targets are the same as $C_{k,\theta }=\sigma_{\theta }^{2}I_{2},\forall k$. The self-positioning error covariance is $C_{n,s}=\sigma_{s}^{2}I_{3},n=1,\cdots ,N$.

### 4.1. Impact of Self-Positioning Error and Angle Measurement Error

We first study the impacts of sensor positioning error and angle measurement error in the proposed intersection method. In order to make a comparison between the intersection method, the LS algorithm [19] and the WLS algorithm [6], we first consider the positioning accuracy of target #1 with the two sensors at t=0 s.

In order to analyze the positioning performance of the concerned algorithms, we measure the performance with the average root mean square error (RMSE), defined for a target, e.g., target #1, by

(99) $$\mathrm{RMSE}=\sqrt{\frac{1}{N_{s}}\sum_{k=1}^{N_{s}}{∥{\hat{g}}_{1}\left(k\right)-g_{1}^{o}∥}^{2}}$$

where ${\hat{g}}_{1}\left(k\right)$ denotes the estimate of the target position at the *k*th simulation run for the target #1, and $N_{s}$ denotes the number of simulation runs.

**Figure 2:**
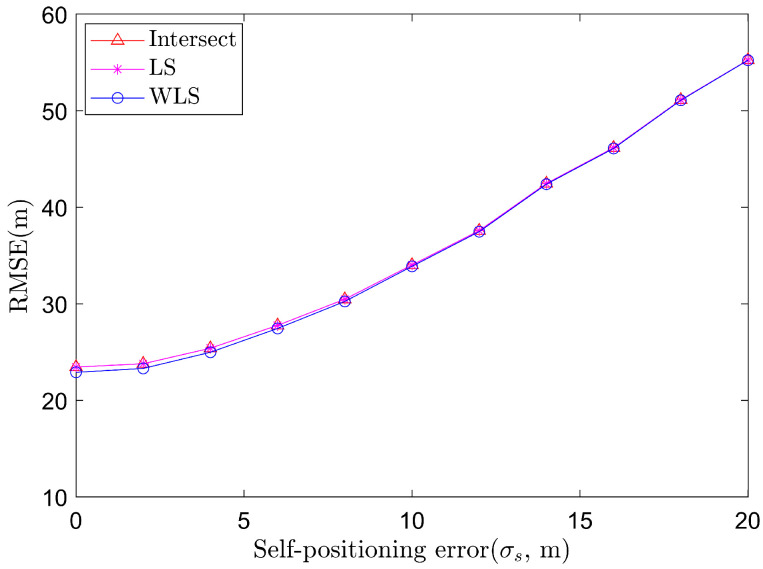
Average RMSE of target localization algorithms based on angle measurements of two sensors at a fixed angle measurement error.

With $N_{s}=$ 10,000 Monte Carlo runs, the RMSE of different self-positioning errors are shown in Figure 2, under the assumption that the covariance matrix is $C_{1,s}=\sigma_{s}^{2}I_{3}$, where we always set $\sigma_{\theta }=0.03^{\circ }$ in this simulation. It can be seen that as $\sigma_{s}$ rises from 0 to 20 m, the three algorithms have close performances and the WLS performs better, under the assumption that the covariance matrices of $C_{k,\theta },k=1,2,3$ and $C_{n,s},n=1,2$ are known exactly.

**Figure 3:**
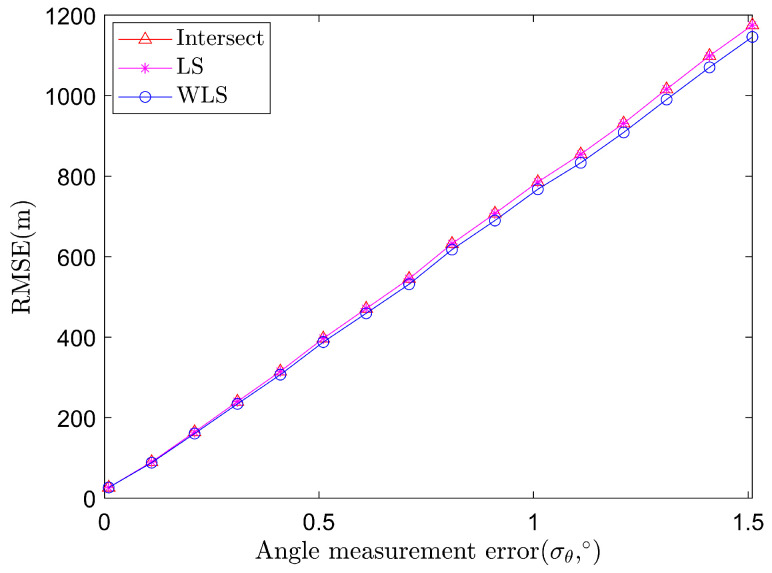
Average RMSE of target localization algorithms based on angle measurements of two sensors at a fixed self-positioning error.

The impacts of the angle measurement error are shown in Figure 3, where $\sigma_{\theta }$ raises from $0^{\circ }$ to $1.5^{\circ }$, where we set $\sigma_{s}=5$ m in this simulation. It shows that the three algorithms have close performances and the intersection method and the LS method perform a little worse than the WLS method. The rise of both the self-positioning error and the angle measurement error will cause the rise of the target positioning error. However, with a better weighting, the WLS algorithm often performs better than the intersection method and the LS method.

### 4.2. Data Association Performance

Consider the parameters of the two sensors and the targets, as shown in Table 1. As the targets are moving in this scenario, we set the sampling interval to 0.1 s. At each sampling instant, each sensor has three measurements corresponding to the three targets. Therefore, the two sensors will totally have nine measurement combinations at each sampling instant, of which three combinations are correct. In data association, we set $p_{f}=1\%$ and then the probability of correct association $p_{c}=99\%$.

**Figure 4:**
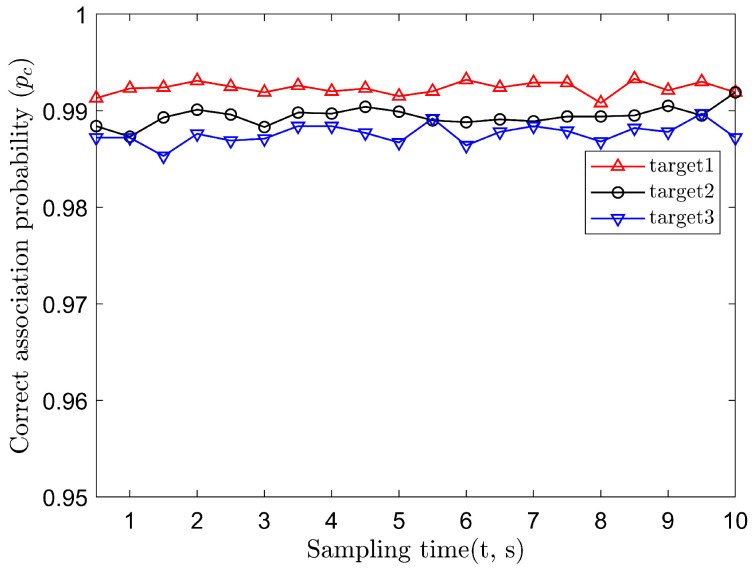
Probability of correct association.

With $N_{s}=2000$ Monte Carlo runs, the averaged correct association probability is shown in Figure 4, where the self-positioning error is $\sigma_{s}=5$ m, and the angle measurement error is $\sigma_{\theta }=0.03^{\circ }$. It can be seen that during the whole sampling period, the correct association probability of the three targets is close to the present value 99%, namely 20 wrong combinations, on average, for each instant.

**Figure 5:**
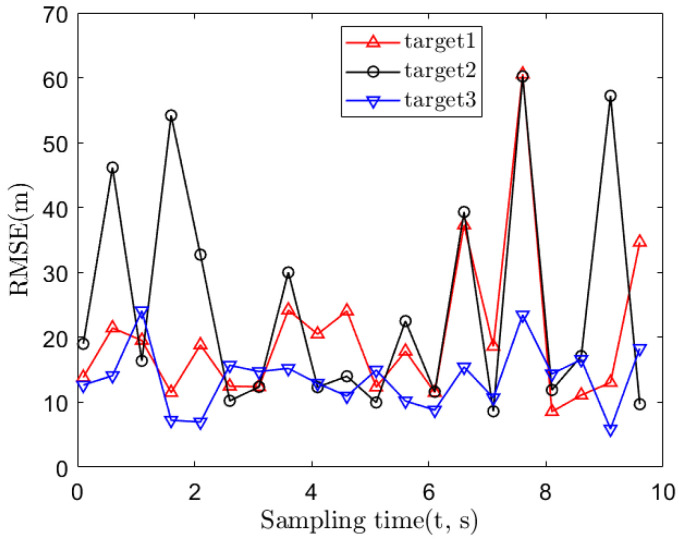
The localization error of the three targets.

Next, we consider the location performance with observations after the data association operation. The RMSEs of the algorithms over the three targets based on the proposed method are shown in Figure 5. In Figure 5, the RMSEs of the three targets are 23.43, 30.05 and 14.45 m. Note that the data association error may affect the localization performance in this case. From Figure 5, the relative position of the sensor and the target will affect the positioning performance, so the impact of the geometry of sensors and targets on the positioning performance will be studied subsequently.

### 4.3. Impact of Target-Sensor Geometry

**Figure 6:**
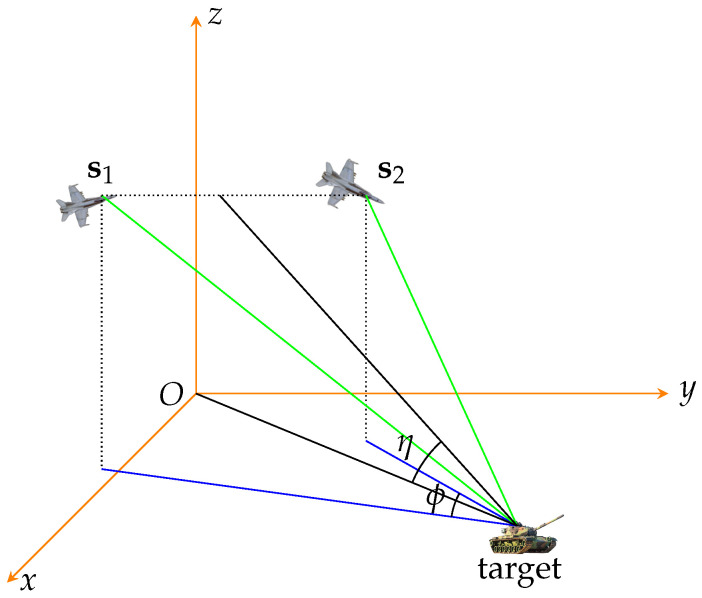
Definition of the intersection angle ϕ and pitch angle η of the plane.

The geometry will play an important role in the positioning performance. Consider the scenario shown in Figure 6, where a target is probed with two passive sensors and the intersection angle of two azimuth lines is denoted by ϕ. In the 3D scenario, we also define a new elevation angle η for convenience and the distance of the target is the same for both the sensors, i.e., 8000 m.

It is assumed that the positions of the two sensors are accurately measured and target #1 can be detected by both two sensors. The two sensors are symmetrically distributed on both sides of target #1. We explore the impact of geometry on the positioning accuracy by changing ϕ and η. In order to illustrate that the errors caused by geometry on the three coordinate axes are different, we define Δx, Δy and Δz to represent the RMSE on the three coordinate axes, respectively, which can be expressed by

(100) $$\begin{array}{c} \Delta x=\sqrt{\frac{1}{N_{s}}\sum_{k=1}^{N_{s}}{∥{\hat{x}}_{1,g}\left(k\right)-x_{1,g}^{o}∥}^{2}} \end{array}$$

(101) $$\begin{array}{c} \Delta y=\sqrt{\frac{1}{N_{s}}\sum_{k=1}^{N_{s}}{∥{\hat{y}}_{1,g}\left(k\right)-y_{1,g}^{o}∥}^{2}} \end{array}$$

(102) $$\begin{array}{c} \Delta z=\sqrt{\frac{1}{N_{s}}\sum_{k=1}^{N_{s}}{∥{\hat{z}}_{1,g}\left(k\right)-z_{1,g}^{o}∥}^{2}}. \end{array}$$

**Figure 7:**
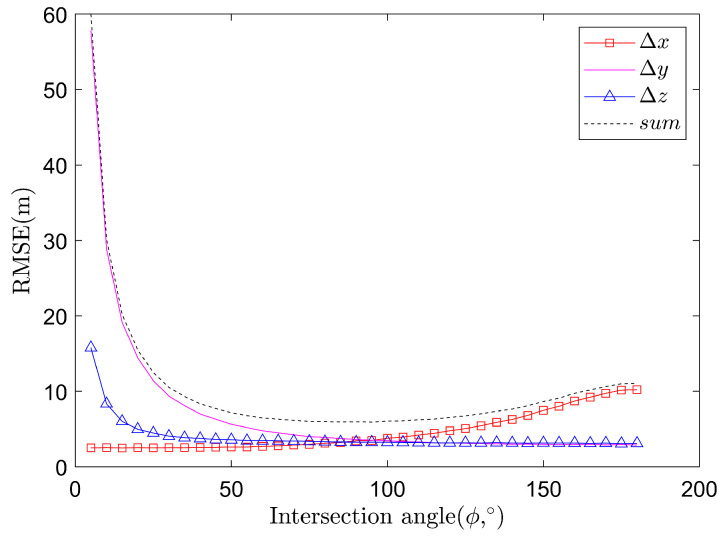
Relationship between localization error and intersection angle ϕ.

With $N_{s}$ = 10,000 Monte Carlo runs, the Δx, Δy, Δz and the spatial error sum denoted by $sum=\sqrt{\Delta x^{2}+\Delta y^{2}+\Delta z^{2}}$ are shown in Figure 7, under the assumption that $\eta =15^{\circ }$, and the angle measurement error $\sigma_{\theta }=0.03^{\circ }$. It can be seen that when the intersection angle ϕ is changed, the positioning error Δx, Δy, Δz are different. When the intersection angle ϕ is equal to $82.58^{\circ }$, the spatial error sum is the best, about 5.58 m, and Δx, Δy and Δz are approximately equal to each other. In fact, the angle around $\phi =90^{o}$ will all lead to high accuracy.

**Figure 8:**
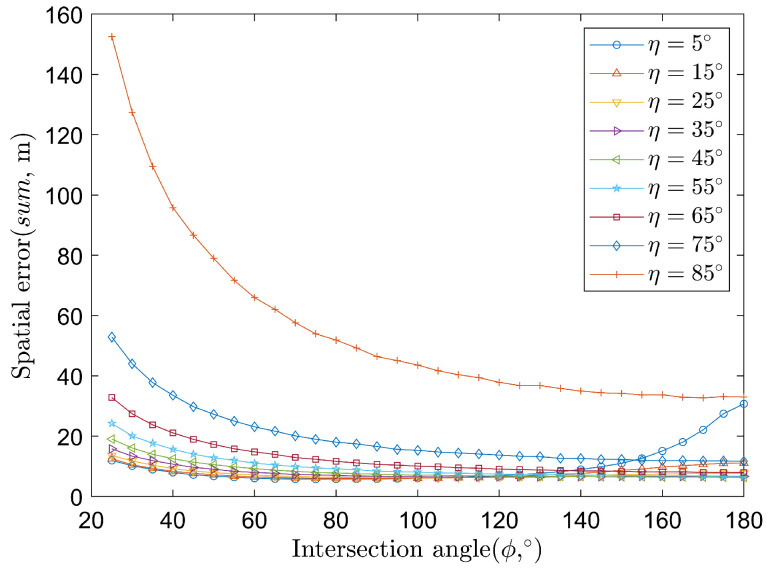
The relationship between spatial error sum and intersection angle ϕ, elevation angle η.

The impacts of intersection ϕ and η on spatial error sum are shown in Figure 8. For η in the range of $0^{\circ }$ to $45^{\circ }$ and ϕ in the range of $25^{\circ }$ to $175^{\circ }$, the spatial error sum is less than 20 m. Therefore, in practice, one can look for geometry with sensors and targets nearly perpendicular to each other to improve the positioning performance.

## 5. Conclusions

This paper studies the data association and signal source localization problems with distributed passive sensors with angle-only observations. A geometry-based data association method is considered, and the concept is that real targets will contribute observations with a small minimal distance. The statistical distribution of the minimal distance of two lines associated with the same target is formulated, based on which a data association method based on hypothesis testing is also developed. The decision threshold is formulated. Meanwhile, for observations that are classified into the same class, three positioning algorithms are studied, namely the intersection method, the LS method and the WLS method. Two kinds of measurements errors are considered, namely sensor self-positioning error and angle measurement error.

In numerical results, we analyze the data association performance of the concerned positioning algorithms and the signal source positioning performance in different scenarios, indicating that the data association algorithm works well and the positioning performances of the algorithms are very close to each other. Meanwhile, if the observation lines are approximately perpendicular to each other, then the localization performance is more accurate.

This algorithm can be used in laser, infrared, and other passive sensors with angle-only measurements. In practice, other information, such as range, ground surface and sea surface, may be available, which can be incorporated into the positioning and association algorithms to improve performance.

## Figures and Tables

**Table 1:** Positions and velocities of sensors and targets.

	Position (m) at t=0 s	Velocity (m/s)	Position (m) at t=10 s
Sensor #1	$s_{1}^{o}\left(0\right)={\left[0,0,0\right]}^{T}$	${\left[50,100,0\right]}^{T}$	$s_{1}^{o}\left(10\right)={\left[500,1000,0\right]}^{T}$
Sensor #2	$s_{2}^{o}\left(0\right)=$ [12,000, 10,000, −800${]}^{T}$	${\left[50,100,0\right]}^{T}$	$s_{2}^{o}\left(10\right)=$ [12,500, 11,000, −800${]}^{T}$
Target #1	$g_{1}^{o}\left(0\right)=$ [18,000, 12,000, 8000]^T^	${\left[20,30,0\right]}^{T}$	$g_{1}^{o}\left(10\right)=$ [18,200, 12,300, 8000${]}^{T}$
Target #2	$g_{2}^{o}\left(0\right)=$ [15,000, 13,000, 7000${]}^{T}$	${\left[20,30,0\right]}^{T}$	$g_{2}^{o}\left(10\right)=$ [15,200, 13,300, 7000${]}^{T}$
Target #3	$g_{3}^{o}\left(0\right)=$ [13,000, 12,000, 5000${]}^{T}$	${\left[20,30,0\right]}^{T}$	$g_{3}^{o}\left(10\right)=$ [13,200, 12,300, 5000${]}^{T}$

## Data Availability

This study did not report any data.

## Appendix A

In this section, we derive the partial derivative vectors $\frac{\partial r}{\partial \theta_{i,m}}$ and $\frac{\partial r}{\partial \theta_{j,m}}$ from (47) and (48). The elements in vector $\frac{\partial r}{\partial \theta_{i,m}}$ and $\frac{\partial r}{\partial \theta_{j,m}}$ can be expressed by

(A1) $$\begin{array}{cc} \frac{\partial r}{\partial \theta_{i,m}} & ={\left(\frac{\partial r}{\partial e_{i}}\right)}^{T}\frac{\partial e_{i}}{\partial \theta_{i,m}}=-\sin\theta_{i,m}\cos\varphi_{i,m}\frac{\partial r}{\partial e_{i,x}}+\cos\theta_{i,m}\cos\varphi_{i,m}\frac{\partial r}{\partial e_{i,y}} \end{array}$$

(A2) $$\begin{array}{cc} \frac{\partial r}{\partial \varphi_{i,m}} & ={\left(\frac{\partial r}{\partial e_{i}}\right)}^{T}\frac{\partial e_{i}}{\partial \varphi_{i,m}} \end{array}$$

(A3) $$\begin{array}{cc} \; & =-\cos\theta_{i,m}\sin\varphi_{i,m}\frac{\partial r}{\partial e_{i,x}}-\cos\theta_{i,m}\sin\varphi_{i,m}\frac{\partial r}{\partial e_{i,y}}+\cos\varphi_{i,m}\frac{\partial r}{\partial e_{i,z}} \end{array}$$

(A4) $$\begin{array}{cc} \frac{\partial r}{\partial \theta_{j,m}} & ={\left(\frac{\partial r}{\partial e_{j}}\right)}^{T}\frac{\partial e_{j}}{\partial \theta_{j,m}}=-\sin\theta_{j,m}\cos\varphi_{j,m}\frac{\partial r}{\partial e_{j,x}}+\cos\theta_{j,m}\cos\varphi_{j,m}\frac{\partial r}{\partial e_{j,y}} \end{array}$$

(A5) $$\begin{array}{cc} \frac{\partial r}{\partial \varphi_{j,m}} & ={\left(\frac{\partial r}{\partial e_{j}}\right)}^{T}\frac{\partial e_{j}}{\partial \varphi_{j,m}}\; \end{array}$$

(A6) $$\begin{array}{cc} \; & =-\cos\theta_{j,m}\sin\varphi_{j,m}\frac{\partial r}{\partial e_{j,x}}-\cos\theta_{j,m}\sin\varphi_{j,m}\frac{\partial r}{\partial e_{j,y}}+\cos\varphi_{j,m}\frac{\partial r}{\partial e_{j,z}} \end{array}$$

where

(A7) $$\begin{array}{cc} \frac{\partial r}{\partial e_{i,x}} & =X^{-\frac{1}{2}}\left(e_{j,y}z_{i,j}-e_{j,z}y_{i,j}\right)-X^{-\frac{3}{2}}Y\left(e_{j,y}^{2}e_{i,x}+e_{j,z}^{2}e_{i,x}-e_{i,y}e_{j,y}e_{j,x}-e_{i,z}e_{j,z}e_{j,x}\right) \end{array}$$

(A8) $$\begin{array}{cc} \frac{\partial r}{\partial e_{i,y}} & =X^{-\frac{1}{2}}\left(e_{j,z}x_{i,j}-e_{j,x}z_{i,j}\right)-X^{-\frac{3}{2}}Y\left(e_{j,z}^{2}e_{i,y}+e_{j,x}^{2}e_{i,y}-e_{i,z}e_{j,z}e_{j,y}-e_{i,x}e_{j,x}e_{j,y}\right) \end{array}$$

(A9) $$\begin{array}{cc} \frac{\partial r}{\partial e_{i,z}} & =X^{-\frac{1}{2}}\left(e_{j,x}y_{i,j}-e_{j,y}x_{i,j}\right)-X^{-\frac{3}{2}}Y\left(e_{j,y}^{2}e_{i,z}+e_{j,x}^{2}e_{i,z}-e_{i,y}e_{j,y}e_{j,z}-e_{i,x}e_{j,x}e_{j,z}\right) \end{array}$$

(A10) $$\begin{array}{cc} \frac{\partial d}{\partial e_{j,x}} & =X^{-\frac{1}{2}}\left(e_{i,z}y_{i,j}-e_{i,y}z_{i,j}\right)-X^{-\frac{3}{2}}Y\left(e_{i,y}^{2}e_{j,x}+e_{i,z}^{2}e_{j,x}-e_{i,y}e_{j,y}e_{i,x}-e_{i,z}e_{j,z}e_{i,x}\right) \end{array}$$

(A11) $$\begin{array}{cc} \frac{\partial d}{\partial e_{j,y}} & =X^{-\frac{1}{2}}\left(e_{i,x}z_{i,j}-e_{i,z}x_{i,j}\right)-X^{-\frac{3}{2}}Y\left(e_{i,z}^{2}e_{j,y}+e_{i,x}^{2}e_{j,y}-e_{i,z}e_{j,z}e_{i,y}-e_{i,x}e_{j,x}e_{i,y}\right) \end{array}$$

(A12) $$\begin{array}{cc} \frac{\partial d}{\partial e_{j,z}} & =X^{-\frac{1}{2}}\left(e_{i,y}x_{i,j}-e_{i,x}y_{i,j}\right)-X^{-\frac{3}{2}}Y\left(e_{i,y}^{2}e_{j,z}+e_{i,x}^{2}e_{j,z}-e_{i,y}e_{j,y}e_{i,z}-e_{i,x}e_{j,x}e_{i,z}\right) \end{array}$$

(A13) $$\begin{array}{cc} Y & =\left|\begin{array}{ccc} x_{i,j} & y_{i,j} & z_{i,j}\\ e_{i,x} & e_{i,y} & e_{i,z}\\ e_{j,x} & e_{j,y} & e_{j,z} \end{array}\right|\; \end{array}$$

(A14) $$\begin{array}{cc} X & ={\left|\begin{array}{cc} e_{i,y} & e_{i,z}\\ e_{j,y} & e_{j,z} \end{array}\right|}^{2}+{\left|\begin{array}{cc} e_{i,z} & e_{i,x}\\ e_{j,z} & e_{j,x} \end{array}\right|}^{2}+{\left|\begin{array}{cc} e_{i,x} & e_{i,y}\\ e_{j,x} & e_{j,y} \end{array}\right|}^{2}\; \end{array}$$

where ${\left[x_{i,j},y_{i,j},z_{i,j}\right]}^{T}=s_{j}-s_{i}$.
